# Osh6 Revisited: Control of PS Transport by the Concerted Actions of PI4P and Sac1 Phosphatase

**DOI:** 10.3389/fmolb.2021.747601

**Published:** 2021-10-12

**Authors:** Andrea Eisenreichova, Bartosz Różycki, Evzen Boura, Jana Humpolickova

**Affiliations:** ^1^ Institute of Organic Chemistry and Biochemistry of the Czech Academy of Sciences, Prague, Czechia; ^2^ Institute of Physics, Polish Academy of Sciences, Warsaw, Poland

**Keywords:** oxysterol-binding protein–related proteins, OSH6, phosphatidylinositol 4-phosphate, phosphatidylserine, phosphatidylinositol (4, 5)-bisphosphate, lipid transport, SAC1

## Abstract

Osh6, a member of the oxysterol-binding protein–related protein (ORP) family, is a lipid transport protein that is involved in the transport of phosphatidylserine (PS) between the endoplasmic reticulum (ER) and the plasma membrane (PM). We used a biophysical approach to characterize its transport mechanism in detail. We examined the transport of all potential ligands of Osh6. PI4P and PS are the best described lipid cargo molecules; in addition, we showed that PIP2 can be transported by Osh6 as well. So far, it was the exchange between the two cargo molecules, PS and PI4P, in the lipid-binding pocket of Osh6 that was considered an essential driving force for the PS transport. However, we showed that Osh6 can efficiently transport PS along the gradient without the help of PI4P and that PI4P inhibits the PS transport along its gradient. This observation highlights that the exchange between PS and PI4P is indeed crucial, but PI4P bound to the protein rather than intensifying the PS transport suppresses it. We considered this to be important for the transport directionality as it prevents PS from returning back from the PM where its concentration is high to the ER where it is synthesized. Our results also highlighted the importance of the ER resident Sac1 phosphatase that enables the PS transport and ensures its directionality by PI4P consumption. Furthermore, we showed that the Sac1 activity is regulated by the negative charge of the membrane that can be provided by PS or PI anions in the case of the ER membrane.

## Introduction

In the past few years, the emerging interest in lipid transport proteins shed some light on the biology of the oxysterol-binding protein (OSBP)-related protein (ORP) family (Olkkonen, 2013; Olkkonen and Li, 2013). ORPs have been identified as lipid transporters that are responsible for transferring specific lipids such as phosphatidylserine (PS) or cholesterol/ergosterol from the site of their synthesis, usually the ER, to their respective acceptor membrane. The mechanism which allows such a selective lipid transport across the concentration gradient relies on phosphoinositides (PPIns), mostly phosphatidylinositol 4-phosphate (PI4P) (von Filseck et al., 2015b).

It has been postulated that the main feature of the transport mechanism is the exchange of the cargo lipid molecule at the target membrane for the PPIn molecule which, upon delivery to the ER, is hydrolyzed, providing the PPIn gradient as a driving force (energy) for the transport against the concentration gradient. This has been first shown elegantly for the yeast ergosterol transporter Osh4 (de Saint-Jean et al., 2011; von Filseck et al., 2015b) and later for the yeast PS transporters Osh6 and Osh7 (Maeda et al., 2013; von Filseck et al., 2015a), as well as for the human cholesterol transporter OSBP (Mesmin et al., 2017).

To maintain the phosphoinositide gradient in live cells, three processes are required: i) PI transport to the PM, ii) its conversion to PI4P by phosphatidylinositol 4-kinases, and iii) PI4P hydrolysis in the ER membrane back to PI carried out by the Sac1 phosphatase (Cai et al., 2014; Chung et al., 2015) that has an essential role in the transport process (von Filseck et al., 2015b).

In this study, we focused on the lipid-binding features of the best characterized PS transporter, the yeast protein Osh6. We compared its binding preferences (relative affinities) toward PS, PI4P, and also to phosphatidylinositol (4,5)-bisphosphate (PIP2) as their relative affinities are critical for defining the directionality of transport. We clearly demonstrated that PI4P is a significantly stronger binder than PS. This protects PS from being transported along the gradient from the PM back to ER. This affinity difference is a defining feature as it keeps the PS plasma membrane level high compared to the level in the endoplasmic reticulum (ER) and prevents unproductive lipid shuffling between the two membranes. We also showed that Sac1 is indispensable for preventing PI4P rebinding to the Osh6 binding pocket and demonstrated how Sac1 allosteric regulation by PS (Zhong et al., 2012) contributes to the transport regulation.

## Materials and Methods

### Protein Expression and Purification

All expression vectors were constructed using restriction cloning. The genes for C2_Lact_-CFP, C2_granuphilin_-CFP, and SidC-CFP were cloned into a modified pHIS-2 vector with an N-terminal 6x-His tag followed by a tobacco etch virus (TEV) protease cleavage site and a CFP coding sequence. The gene for Osh6 was cloned into a pRSFD (Novagen) vector with an N-terminal His6x-GB1 solubility tag and a TEV cleavage site. Mutation to produce catalytically dead Sac1(C392S) was generated by site-directed mutagenesis. The proteins, including the C-terminal His-tagged Sac1 and Sac1(C392S), were expressed in ZYP-5052 autoinduction media in *Escherichia coli* BL21 Star cells using our standard protocols (Chalupska et al., 2017; Klima et al., 2017). Briefly, the cells were harvested by centrifugation, resuspended in a lysis buffer (50 mM Tris pH 8, 300 mM NaCl, 20 mM imidazol, and 3 mM β-mercaptoethanol), and lysed by sonication. After the lysate was cleared by centrifugation and incubated with a nickel-chelating resin (Machery-Nagel), the proteins were eluted with the lysis buffer supplemented with 300 mM imidazol. The hexahistidine tag or GB1 tag of SidC-CFP, C2_Lact_-CFP, C2_granuphilin_-CFP, and Osh6 was cleaved off with TEV protease. When required, the proteins were purified by cation exchange (Osh6) and anion exchange (Sac1 and Sac1 (C392S)). The final purification step of all proteins comprised exclusion chromatography on HiLoad 16/600 Superdex 75 pg (Cytiva) in 20mM Tris pH = 7.4, 300 mM NaCl, and 3mM β-mercaptoethanol. Purified recombinant proteins were stored at −80°C.

### Preparation of LUVs

Large unilamellar vesicles (LUVs) were prepared as before (Humpolickova et al., 2017). Briefly, stock solutions of lipids and labeled lipid analogs in organic solvents (chloroform, methanol) were mixed in the required ratio. The organic solvents were evaporated in the stream of dry nitrogen, and the lipid films were placed into the vacuum chamber for an hour to remove all the traces of the original solvents. The films were than resuspended in the LUV buffer (10 mM TRIS, pH = 7.4, 150 mM NaCl, 3 mM β-mercaptoethanol, and 1 mM EDTA). The solution of multilamellar membranes was homogenized in an extruder (Avestin, Ottawa, Canada) through the membrane with 50 or 100 nm pores. The total concentration of lipids in the LUV buffer was 1 mM.

### FCCS Microscopy

The FCCS experiments were acquired on a confocal microscope LSM 780 (Zeiss, Hamburg, Germany). The microscope was equipped with two external SPAD detectors and a FLIM upgrade kit (Hydraharp, Picoquant, Berlin, Germany), which was used for data collecting. The excitation light was guided to the water immersion objective (× 40, N.A. = 1.2), and the signal produced after it had been spatially filtered by a pinhole which was split on the two SPAD detectors with 482/35 and 679/41 emission filters placed in front of them. We used 458 nm argon laser line (continuous wave—cw) to excite CFP and 625 nm line of the In tune laser (pulsed, 40 MHz) to excite DiD. The data were correlated using the homemade scripts in MATLAB (Mathworks, Nattick, MA) (Wahl et al., 2003), and the different TCSPC patterns resulting from the combination of the cw and pulsed excitation were used for eliminating the bleed-through signal (Skerle et al., 2020).

### FCCS Assays

In both PI4P and PIP2 transport assays, 10 µl of PI4P/PIP2-containing LUVs were mixed with 40 µl of LUVs that served as the target for PI4P/PIP2 and that also contained the lipid analog DiD (DiD/lipid = 1/10,000). A biosensor (SidC-CFP and C2_granuphilin_-CFP) was added so that its concentration in the 200 µl total volume was 100 nM. Short (200 s) FCCS experiment was acquired prior to the addition of Osh6 (1 µM final concentration). Immediately upon Osh6 addition, the measurement was set for additional 30 min. For the transport of either PI4P or PIP2, the LUVs contained 5 mol% of the particular phosphoinositide; for the experiments following the transport of either PI4P or PIP2 in the mixture with the other phosphoinositide, the LUVs contained 5 mol% of each. The PS content in the acceptor LUVs scales from 0 to 20 mol%.

To address the effect of the Sac1 participation, the PI4P-accepting membrane contained 5 mol% of DGS-NTA(Ni), which assures the Sac1 attachment *via* its C-terminal His-tag. The concentration of either wild-type Sac1 or C392S mutant in the experiment was 1 µM.

In the assay that addresses the Sac1 phosphatase activity, LUVs contained 0.1 mol% of PI4P (to keep the changes in the dynamic range of the biosensor), 5 mol% of DGS-NTA(Ni), and the 20 mol% of the negatively charged lipid (PS, PI, and PG), and the lipid analog DiD (DiD/lipid = 1/10,000). 40 µl of LUVs were mixed with SidC-CFP (final concentration: 100 nM), Sac1 (final concentration: 1 µM), and the LUV buffer to final volume of 200 µl. Upon Sac1 addition, the measurement was run for 30 min.

All the FCCS experiments were acquired at least in three independent experiments to make sure that the observed trends are reproducible and robust.

### FRET Assay

In a PS transport assay, 100 μl of donor LUVs containing 90 mol% POPC, 5 mol% POPS, and 5 mol% DGS-NTA(Ni) were mixed with 100 μl of acceptor LUVs. The acceptor LUVs consisted of 95% POPC and 5 mol% ATTO488-DOPE. To monitor the effect of phosphoinositides on the PS transport, the acceptor LUVs contained additional 5 mol% PI4P or PIP2. A fluorescence biosensor for PS, C2_Lact_-CFP, was added to the final concentration of 125 nM (in the final volume of 800 μl). Shortly after the start of the measurement, Osh6 (100 nM) was injected. When Sac1 or Sac1(C392S) (200 nM) was present in the transport, it was first incubated for 5 min with the donor LUVs to allow its binding to DGS-NTA(Ni). PS transport was monitored by measuring the FRET between C2_Lact_-CFP and ATTO488-DOPE at 527 nm upon excitation at 400 nm. The measurement was run for 30 min on a Fluoromax 4 spectrophotometer (Horiba, Kyoto, Japan). The FRET kinetics was measured in at least three independent sets to prove the reproducibility and robustness of the results.

### Choice of the Assay

In our study, we used different assays for different cargos. In the case of PS transport, we have realized that the C2_Lact_-CFP response depends on the presence of PPIns in the PS-accepting membrane. The biosensor was less sensitive to PS in PPIn’s absence. This significantly complicated the FCCS approach as it was almost blind to small amounts of PS in the accepting membrane devoid of PPIns. The FRET approach was more robust, and its readout was insensitive to PPIns. On the contrary, using the FRET approach for PPIns that are less transported than PS gives small overall readout with significant noise. Altogether, we paid attention to i) whether the readout monitors the level of the evaluated cargo independently on other lipids significant for the transport process, and ii) to the extent and quality of the readout change.

### GUV Preparation and Imaging

Giant unilamellar vesicles (GUVs) were prepared as before (Boura and Hurley, 2012; Dubankova et al., 2017). Lipids were mixed in the desired ratio in chloroform so that their final concentration was 5 µg/µl. 2 × 9 µl was spread on two ITO-coated glass electrodes that were plane parallely assembled into a Teflon chamber. Five ml of 600 mM sucrose was added, and a sinusoidal voltage of 10 kHz frequency and an amplitude of 1 V were applied on them for 1 hour at 60°C. After that, 50 µl of donor and 50 µl of acceptor GUVs were mixed with 100 µl of isosmotic buffer (25 mM Tris pH 8, 10 mM MgCl_2_, 20 mM imidazole, 261.5 mM NaCl, and 2 mM βME) containing the appropriate biosensor (70 nM) and Osh6 (100 nM). The mixture was placed on the BSA-coated cover glass (bottom of the 4-chamber dish), and the images were taken.

The images were acquired on a Leica SP8 confocal microscope (Mannheim, Germany) using ×60 water immersion objective lens. To avoid bleed-through, the images were captured in line sequentially. CFP was excited by 458 nm argon laser line, and DiD that was added to the acceptor GUVs was excited by 633 nm He–Ne laser line. Emission light was split onto two HyD detectors according to the emission spectra of the fluorophores.

### Chemicals

All the lipids in this study were purchased from Avanti Polar Lipids (Alabaster, AL). In particular, we used POPC, POPS, brain PI4P, brain PI(4,5)P2, liver PI, POPG, and DGS-NTA(Ni). ATTO488-DOPE was purchased from ATTO-TEC (Siegen, Germany). DiD and other chemicals were purchased from Sigma-Aldrich (St. Louis, MO).

### Molecular Dynamics Simulations

The atomic coordinates of yeast Sac1 were taken from the crystal structure deposited in the Protein Data Bank (PDB) with the entry code of 3LWT (Manford et al., 2010). Monte Carlo (MC) simulations of Sac1 in contact with a planar membrane were performed within the framework of the Kim–Hummer model (Kim and Hummer, 2008), in which both the membrane and solvent are implicit, whereas crystallized protein domains are treated as rigid bodies. Here, Sac1 was taken as a single rigid body as determined by the crystal structure with the PDB code 3LWT. In the course of the MC simulations, the Sac1 rigid structure was subjected to translations and rotations relative to the membrane surface. Simulated annealing was used to determine the minimum of Sac1–membrane interaction energy. The position of the Sac1 rigid structure corresponding to the energy minimum was used as the initial position of Sac1 in MD simulations.

The initial systems for MD simulations were prepared using the Input Generator and the Membrane Builder in the CHARMM-GUI website (Jo et al., 2007; Jo et al., 2008; Wu et al., 2014; Lee et al., 2016). Specifically, two bilayer segments with equal lateral dimensions of 11 by 11 nm were formed independently. In one case, the bilayer was composed of 292 POPC and 72 POPS lipids (i.e., with the molar ratio of about 4:1). In the other case, the bilayer was composed of 352 POPC lipids. In both cases, Sac1 was placed on top of the lipid bilayer in accordance with the minimum energy position obtained from the MC simulations. The lipid–protein systems were solvated next, and sodium and chloride ions were added to neutralize the systems and to reach a physiological ion concentration of 150 mM.

The MD simulations were performed using NAMD 2.10 with CHARMM36 force field and the TIP3P model for water molecules (MacKerell et al., 1998; Klauda et al., 2010; Best et al., 2012; Klauda et al., 2012; Phillips et al., 2020). Temperature was kept at 303 K through a Langevin thermostat with a damping coefficient of 1/ps. Pressure was maintained at 1 atm using the Langevin piston Nose–Hoover method with a damping timescale of 25 fs and an oscillation period of 50 fs. Short-range nonbonded interactions were cutoff smoothly between one and 1.2 nm. Long-range electrostatic interactions were computed using the particle mesh Ewald method with a grid spacing of 0.1 nm. Simulations were performed with an integration time step of 2 fs.

The initial systems for MD simulations were energy-minimized using a conjugate gradient method and then equilibrated in a standard procedure using input files provided by the CHARMM-GUI Membrane Builder (Jo et al., 2007; Wu et al., 2014). Next, for each of the two simulation systems (i.e., POPC-POPS-Sac1 and POPC-Sac1), we performed two production runs of 60 ns each, amounting to 240 ns of MD data for analysis. The trajectories were visualized and analyzed using VMD (Humphrey et al., 1996). To determine contacts between amino acid residues and lipid molecules, we used a simple distance criterion, namely, an amino acid residue was taken to be in contact with a lipid molecule if the minimum distance between non-hydrogen atoms of the residue and the lipid was smaller than 0.45 nm.

## Results

Lipid transport can be observed as pertinent to each of the potential participants, that is, all lipids that can be potentially transported by Osh6. We have examined the transport of both PS and PI4P that have been identified to bind Osh6. In addition, we also tested PIP2 as a potential cargo of the Osh6 molecule.

### PS Transport

**FIGURE 1:**
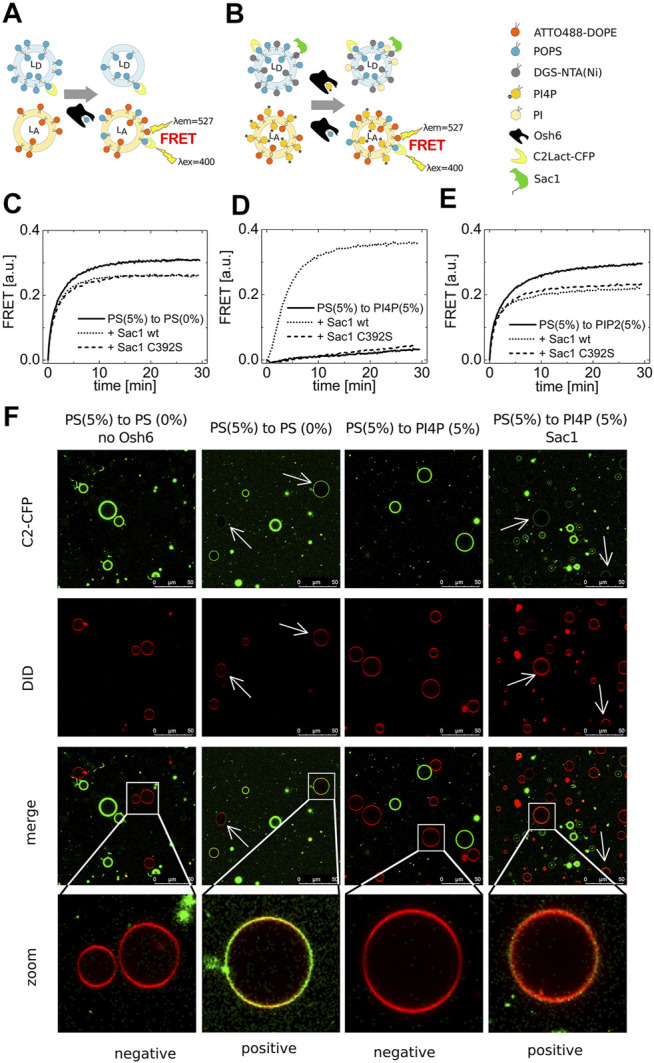
PS transport. **(A)** and **(B)** Schematics of the FRET assay with and without Sac1. Temporal evolution of the PS transport to LUVs composed of POPC solely **(C)**, to LUVs with 5 mol% of PI4P **(D)**, to LUVs with 5 mol% of PIP2 **(E)**. LUVs are either not decorated by Sac1 (solid curves), or are decorated by Sac1 wt (dotted curves), or by Sac1 C392S (dashed curves). **(C–E)** Representative curves out of at least three independent experiments. **(F)** Reconstitution of the PS transport to the membranes of GUVs. Red GUVs are PS-accepting GUVs, and presence of the green PS biosensor on the red GUVs refers to transport.

We examined the transport of PS first because Osh6 was reported to be a PS transporter in several studies (Maeda et al., 2013; von Filseck et al., 2015a). We have utilized a FRET assay to monitor the amount of transported PS in the acceptor membrane by observing the energy transfer between CFP fused to a PS biosensor [C2 domain from Lactadherin C (LactC) ] and ATTO488-DOPE in the acceptor membrane (Figures 1A,B). To validate the utility of this assay, we determined the concentration range of PS at which the assay provides a dynamic response to PS change. These preliminary experiments revealed that the FRET assay can be used between 0 and 2 mol% of PS in the membrane of large unilamellar vesicles (LUVs). PS increases above this level do not cause a further increase in the FRET signal (Supplementary Figure S1).

Having a well-characterized assay in hands, we examined the PS transport to various acceptor membranes differing in the lipid composition. Figure 1C clearly shows that PS transport along a concentration gradient to an acceptor membrane composed only of POPC (and the fluorescence acceptor molecules, ATTO488-DOPE) is readily detectable. Simply viewed, the Osh6 molecules pick up PS molecules from any membrane of high PS content and deliver them to the membrane of low PS content, which is a process simply driven by entropy. If this were to happen in intact cells, it would eliminate the high PS concentration in the PM. We reasoned that PI4P or PIP2, both present in the PM, would inhibit the undesirable backward PS transport between the PM and the ER. To test this, we applied the same amount of PI4P in the PS-accepting membrane and PS in the donating membrane and found that the PS transport was significantly impaired (Figure 1D). Moreover, PIP2 can also bind to the Osh6 transport pocket, but, in contrast to PI4P, the presence of PIP2 in the acceptor membrane inhibits PS transport only slightly (Figure 1E).

We then focused on the role of the Sac1 phosphatase. We tethered Sac1 to the donor PS membrane (Figure 1B) by His-tag on the C-terminus of the cytosolic part of Sac1 that binds to DGS-NTA(Ni) present in the PS-donating membrane. Upon Sac1 attachment, the PS transported to the PI4P-containing membranes can be fully restored (Figure 1D dotted curve). The specificity of this process was demonstrated as the catalytically dead mutant of Sac1(C392S) was without effect (Figure 1D dashed curve). This suggests that the presence of PI4P in the acceptor membrane can keep the PS gradient between the plasma membrane and ER. The hydrolysis of PI4P in the ER membrane by Sac1 shifts the PI4P-binding equilibrium and leads to the release of the transported PI4P molecule and freeing up the lipid-binding pocket of Osh6 for further binding of PS to deliver it to the PM. By the cooperation of PI4P and Sac1, transportation of PS can be effectively regulated. Figures 1C,E show a small drop in the PS transport efficiencies upon decoration of the PS-containing membrane with Sac1 (wt as well as C392S mutant). This may be a result of protein crowding on the membrane, that is, lower access of Osh6 to the cargo.

Also, we reconstituted the PS transport into the membrane of giant unilamellar vesicles (GUVs) (Figure 1F). The PS-accepting GUVs were doped with DiD (red circles), and the PS-donating membranes were labeled by the PS biosensor C2-CFP (green circles). The transport was observed approximately 30 min upon Osh6 addition. The appearance of green color on the originally red membranes refers on the transport process in progress.

### PI4P Transport

**FIGURE 2:**
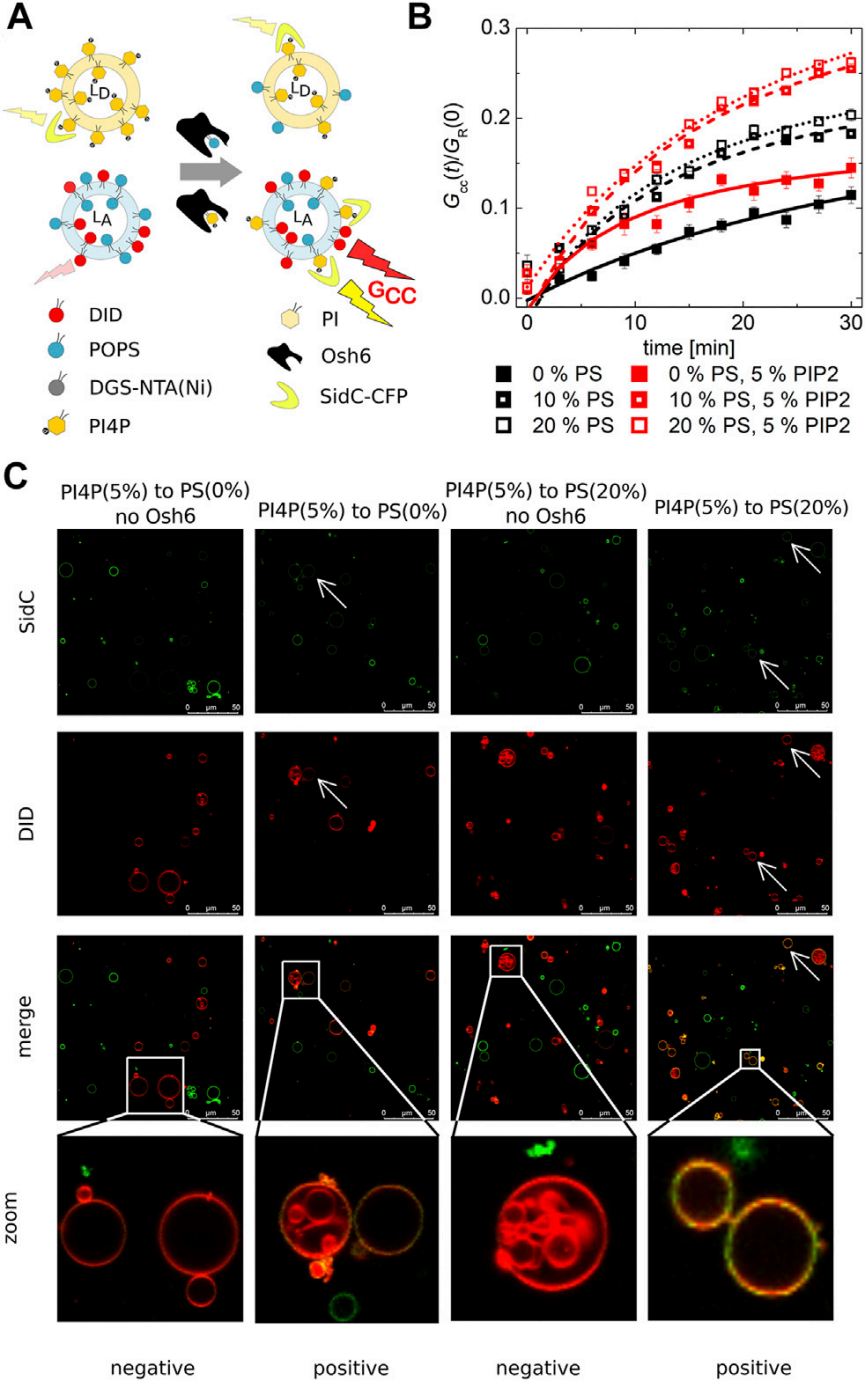
PI4P transport observed by the FCCS assay. **(A)** Schematics of the FCCS assay. **(B)** Temporal evolution of the transport-monitoring parameter *G*_cc_/*G*_R_. PI4P was transported to the LUVs with an increasing amount of PS: POPC only (full squares), 10 mol% of PS (half-filled squares), and 20 mol% of PS (empty squares). The PI4P-donating membrane (5 mol% PI4P) was also enriched in PIP2 (5 mol%): wo/w PIP2—black/red squares. The error bars stand for the standard deviation. **(C)** Reconstitution of the PI4P transport to the membranes of GUVs. Red GUVs are PI4P-accepting GUVs, and the presence of the green PI4P biosensor on the red GUVs refers on the transport. The presented data represent the trends that were observed in minimum three independent experiments.

PI4P transport was monitored by fluorescence cross-correlation spectroscopy (FCCS). Donor LUVs containing PI4P were mixed with PI4P-accepting LUVs of varied lipid composition also containing the fluorescence marker DiD. The PI4P biosensor, SidC-CFP, was localized only at the donor membrane at time zero. Upon Osh6 addition, the biosensor started to move to the acceptor LUVs following the PI4P transport (Figure 2A). As a result of this transport process, we could observe the cross-correlation of the fluorescence signal of CFP and DiD. Importantly, also for this assay, we established its dynamic range which was found to be between 0 and 1 mol% (Supplementary Figure S1) in agreement with the nanomolar-binding affinity of SidC and PI4P (Luo et al., 2015).

We already established that PI4P can efficiently block the PS transport along the gradient, that is, Osh6 significantly favors PI4P binding in comparison to PS. Next, we focused on the PI4P transport and its dependence on the PS concentration in the PI4P-accepting membrane.

Our FCCS analysis revealed that PI4P is also transported along its gradient by Osh6 although the speed of PI4P transport was slower than that of PS (Figure 2B). In the case of the PS transport (Figure 1), saturation of the FRET response happens within few minutes, whereas in the case of SidC that senses PI4P, only small amount of PI4P with a continuous increase appears in the target membrane even after 30 min upon Osh6 addition.

PIP2 is the major phosphoinositide of the plasma membrane (van Meer et al., 2008); we have enriched the PI4P-containing LUVs by PIP2 in the 1–1 M ratio (5 mol% each). However, this alteration did not have any effect on the PI4P transport, suggesting lower affinity of PIP2 to Osh6 than PI4P (Figure 2B red curves).

PI4P transport was also reconstructed in the membranes of GUVs (Figure 2C), PI4P donor GUVs were labeled by the SidC-CFP, and PI4P acceptor GUVs contained the fluorescence marker DiD. The appearance of the double-labeled vesicles visualized approximately 30 min upon Osh6 addition reports on the transport. The visual inspection of the GUVs shows a higher CFP signal in the acceptor GUVs that contained 20 mol% of PS.

We also used the FCCS assay to explore the impact of Sac1 on the PI4P transport. In this setup, Sac1 was again tethered to the PI4P-accepting membrane. We expected to see the result of two tightly linked processes, that is, the transfer of PI4P followed by its hydrolysis in the acceptor membrane. Because FCCS counts the double-labeled LUVs, PI4P accumulation in the acceptor vesicles as a result of its transfer from donor membranes would appear as an increase in the cross-correlation amplitude, whereas PI4P hydrolysis would result in a drop of the cross-correlation amplitude. Altogether, the transfer coupled to consequent hydrolysis shows a smaller increase of the cross-correlation amplitude than the situation when the hydrolysis does not occur.

**FIGURE 3:**
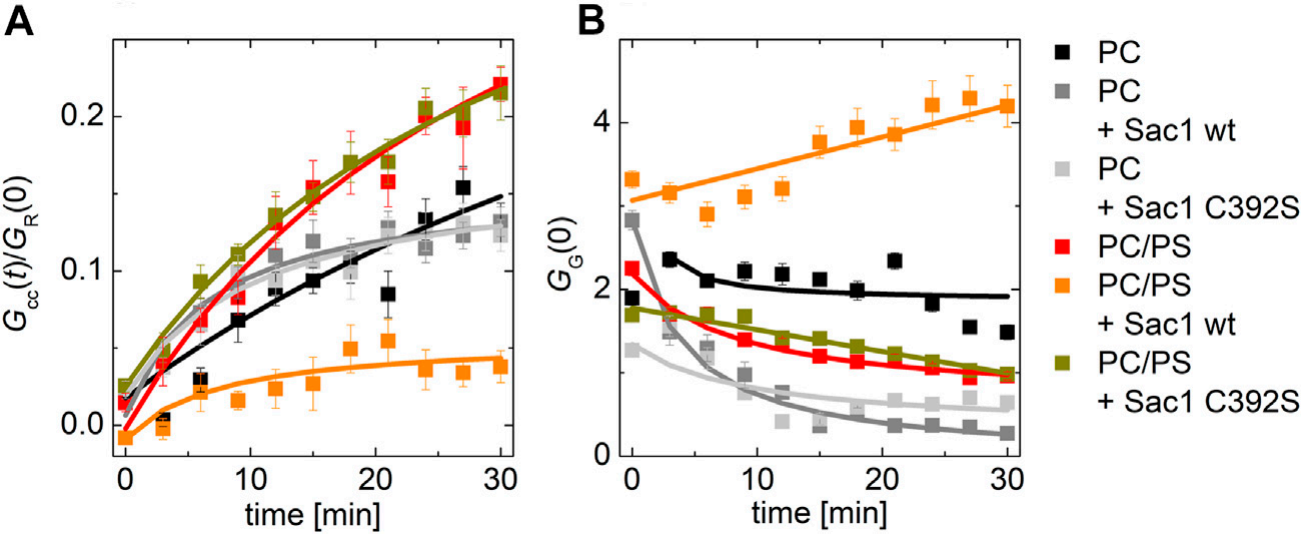
PI4P transport followed by Sac1 dephosphorylation viewed by FCCS. **(A)** The temporal evolution of the transport-monitoring parameter *G*_cc_/*G*_R_ and **(B)** the temporal evolution of the amplitude of the CFP autocorrelation function, and the parameter reporting on the biosensor binding *G*_G_. The PI4P-accepting membrane was composed of POPC and the following: i) not decorated by Sac1 (black squares), ii) decorated by Sac1 wt (dark gray squares), and iii) decorated by Sac1 C392S (light gray squares). The PI4P-accepting membrane was composed of POPC/PS (20 mol% of PS) and the following: i) not decorated by Sac1 (red squares), ii) decorated by Sac1 wt (orange squares), and iii) decorated by Sac1 C392S (dark yellow squares). The error bars stand for the standard deviation. The presented data represent the trends that were observed in minimum three independent experiments.

Remarkably, the effect of Sac1 showed a dependence on the lipid composition of the PI4P acceptor membrane. In a bilayer composed of POPC only, we did not observe any effect of Sac1 (Figure 3A dark gray curve). However, a negatively charged membrane containing 20 mol% PS supported much enhanced Sac1-mediated PI4P hydrolysis (Figure 3A orange curve). In the case of the Sac1 catalytically dead mutant, the amount of transported PI4P was almost identical to that of the control without Sac1 (Figure 3A gray curve) for PC and dark yellow curve for 20 mol% of PS. These results suggest that the presence of PS in the acceptor membrane changes the activity of Sac1 and consequently the elimination of PI4P.

Figure 3B displays the temporal evolution of the autocorrelation amplitude of the CFP signal. The autocorrelation amplitude is inversely proportional to the number of independently moving fluorescent particles (LUV with several dye molecules is seen as one particle) present in the solution. During the PI4P transport, acceptor LUVs were getting enriched in PI4P, leading to an increase of the number of particles recognized by the PI4P biosensor SidC-CFP; as a result, the autocorrelation amplitude lowers (Figure 3B, curves: black, light dark, light gray, red, and dark yellow). After the transported PI4P is hydrolyzed in the acceptor membrane, there are fewer vesicles newly decorated by SidC-CFP, and at the same time, the PI4P level in the donating vesicles lowers, which leads to their lower fluorescence signal, and in the solution, the free PI4P biosensor SidC-CFP eventually appears. This leads to an initial increase in the autocorrelation amplitude (Figure 3B orange curve). The explanation of the amplitude increase is not trivial as also the brightness (number of dye molecules per LUV) of the individually moving fluorescent molecules come into play; however, as explained in SI Figure 2, the rise of the CFP-SidC autocorrelation amplitude fits well to the expected process.

### Regulation of Sac1 Activity

The transport experiments indicate that the catalytic activity of Sac1 is significantly affected by the presence of PS in the membrane where Sac1 is located and PI4P hydrolysis occurs. This observation suggests that the PS in the ER membrane may stimulate Sac1. Therefore, we decided to investigate this process in greater detail. We have examined the hydrolysis of PI4P by Sac1 tethered to membranes of various lipid compositions. We aimed to determine whether the activity is regulated by PS specifically or other negatively charged lipids would act similarly.

For this, we prepared LUVs that contained 0.1 mol% PI4P and 20 mol% of a selected negatively charged lipid (PS, PI, and PG). The amount of PI4P was chosen to be low to match the sensitivity range of our biosensor SidC. The LUVs also contained a fluorescence marker DiD. Upon addition of the recombinant protein Sac1, we monitored teh decrease of the cross-correlation amplitude between the SidC-CFP and the DiD signal.

**FIGURE 4:**
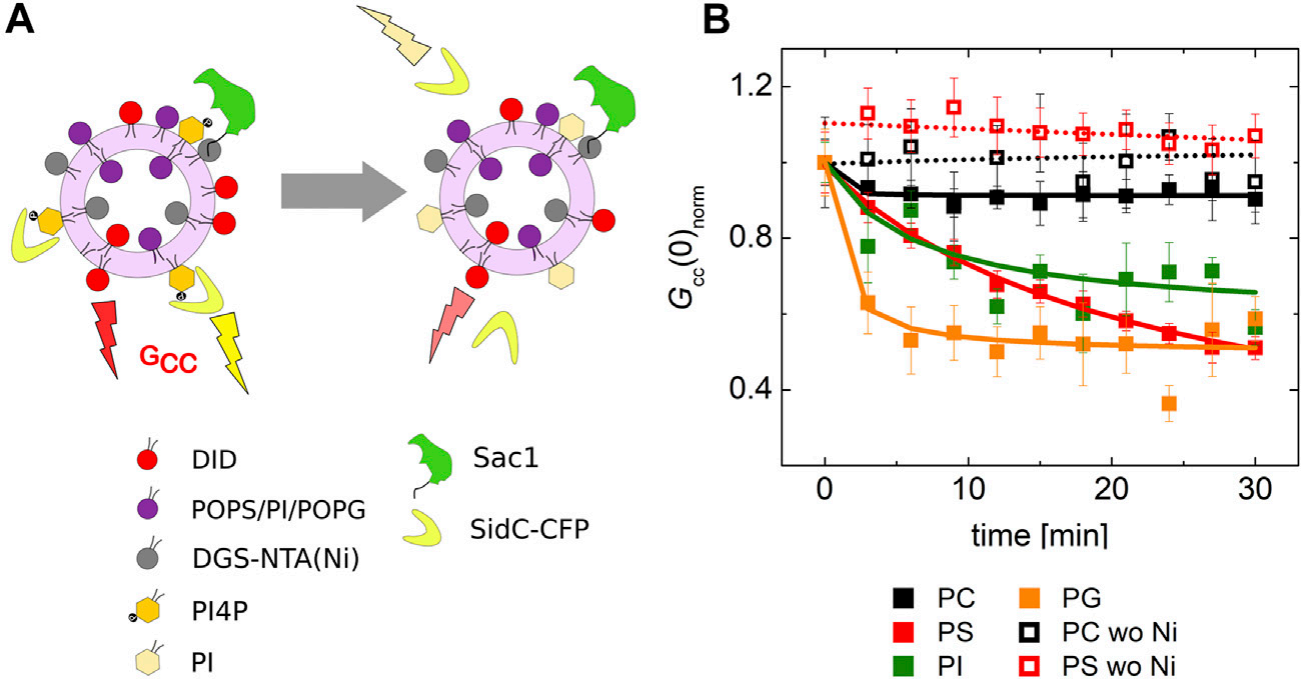
PI4P dephosphorylation by Sac1 followed by FCCS. **(A)** Schematics of the FCCS experiment. **(B)** Temporal evolution of the normalized *G*_cc_ amplitude referring on the SidC-CFP release (PI4P dephosphorylation) from the membranes of LUVs. The membrane where the reaction was followed was composed of POPC (black squares), POPC/PS (20 mol% of PS) (red squares), POPC/PI (20 mol% of PI) (green squares), and POPC/PG (20 mol% of PG) (orange squares). The membrane either contained DGS-NTA(Ni) for Sac1 attachment through His-tag (solid squares), or DGS-NTA(Ni) was omitted and Sac1 remained in the solution (empty squares). The amount of PI4P in all the examined membranes was 0.1 mol%. The error bars stand for the standard deviation. The presented data represent the trends that were observed in minimum three independent experiments.

Our experiment clearly showed that many of the negatively charged lipids probe (PS, PI, and PG) had a similar and significant impact on the activity of the Sac1 enzyme (Figure 4B, solid red, green, orange, and black squares for PS, PI, PG, and PC, respectively). LUVs that were not decorated by Sac1 (Sac1 was just in solution, not tethered to LUVs, and the LUVs did not contain DGC-NTA(Ni) for the His-tag attachment) did not display any significant change of the PI4P level in the membrane (measured by the cross-correlation, Figure 4B, empty squares). However, all the negatively charged lipids significantly supported the reaction. This refers to electrostatic forces that change PI4P hydrolysis by Sac1 rather than a specific PS-related interaction. As the His-tag attachment to the bilayer seems to be crucial for the hydrolysis, the enzyme in our hands has to work mainly in the *cis*-regime (on the same membrane, which it is attached to) (Li and Xu, 2019). The *trans*-regime (acting on the opposing membrane) would be hardly visible under our conditions since even the unattached enzyme does not significantly hydrolyze PI4P. Even though not excluding the *trans*-regime totally—the membranes in cells are more adjacent to each other compared to freely diffusing LUVs—our data support the *cis-*activity of Sac1 in agreement with the study of Zewe et al. (2018).

**FIGURE 5:**
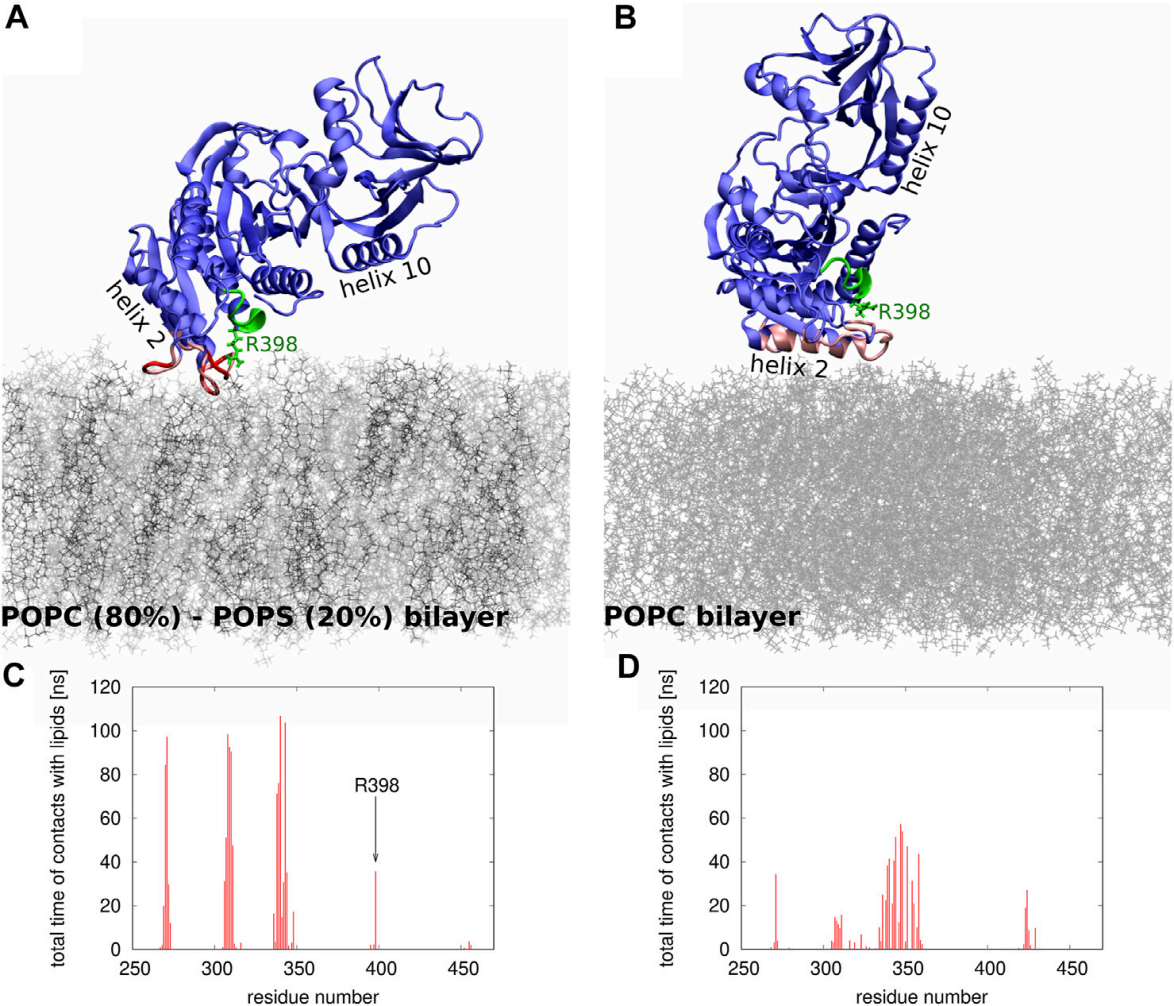
**(A,B)** Snapshots from the MD simulations of Sac1 at (A) POPC-POPS and (B) POPC bilayer. The POPC and POPS molecules are shown as thin sticks in gray and black, respectively. The Sac1 molecule is shown in blue except for the regions that make strong (shown in red) or transient (shown in pink) contacts with lipids. The catalytic P-loop is shown in green. Helices 2 and 10 are indicated to show the differences in how Sac1 is oriented relative to **(A)** POPC-POPS and **(B)** POPC bilayers. Arg 398 is shown in stick representation. **(C,D)** Histograms showing which amino acid residues and how frequently make contacts with **(C)** POPC-POPC and **(D)** POPS bilayers.

To gain molecular insights into the impact of electrostatics on Sac1 interactions with lipid membranes, we performed two series of MD simulations: in the first one, the membrane was composed of POPC (80%) and POPS (20%) lipids, and in the second one, the membrane contained only POPC molecules. Figure 5 illustrates the differences in the positioning of Sac1 at the membrane as observed in the two series of simulations. In the MD simulations with the POPC-POPS bilayer (Figures 5A,C), Sac1 was found to be anchored to the membrane by three loops consisting of amino acid residues 269-273, 306-311, and 336-344. Interestingly, Arg 398 was also found to make transient contacts with the lipids. In contrast, in the MD simulations with the POPC bilayer (Figures 5B,D), Sac1 was observed to vary its orientation relative to the membrane surface, which resulted in more transient contacts of Sac1 with the lipids. The Sac1 regions that were found to make transient contacts with the POPC bilayer were mainly within helix 10 and the loop 336-344. Importantly, Arg 398 did not make any contacts with POPC molecules and was observed to point away from the membrane surface. These differences in the Sac1 position at the POPC-POPS (Figures 5A,C) and POPC (Figures 5B,D) bilayers provide molecular insights into the possible impact of negatively charged lipids on the enzyme activity.

### PIP2 Transport

PIP2 is an important plasma membrane phosphoinositide that can potentially also affect the PS transport process. It has been shown that it indeed can bind the ORD domain of ORP5/8 (Ghai et al., 2017) belonging to the same subfamily. We have examined the ability of Osh6 to transport PIP2. From the variety of described PIP2-specific biosensors (Saad et al., 2006; Wan et al., 2015; Hertel et al., 2020), we chose a C2 domain from granuphilin fused to CFP (C2_granuphilin_-CFP) (Wan et al., 2015) that had a linear response to PIP2 concentration in the range 0–5 mol% of PIP2 (Supplementary Figure S1).

Our assay revealed that PIP2 transport indeed occurs similarly to the transport of PI4P, and it is sensitized by the presence of PS in the PIP2-accepting membrane. PIP2 transport is slower when PI4P also is present in the same membrane, illustrating that PIP2 and PI4P compete for the same ligand-binding pocket of Osh6, or it binds to a different binding site (Wang et al., 2019) where it might regulate the entry of PI4P to the binging pocket. The coexistence of both PPIns in the plasma membrane suggests that both of them could potentially act as a cargo for the PS exchange; however, PI4P would be used preferentially due to its higher affinity for Osh6. Also, as seen in our previous experiments (Figure 1), unlike PI4P, PIP2 cannot significantly prevent PS from being transported along its gradient.

**FIGURE 6:**
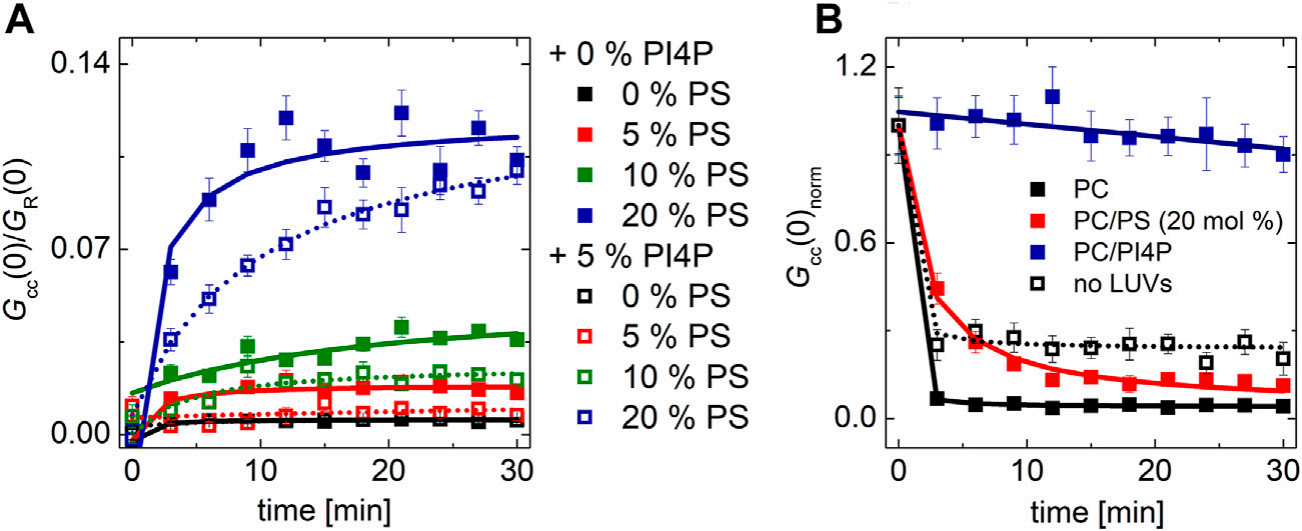
PIP2 transport and extraction. **(A)** Temporal evolution of the transport readout. The PIP2-accepting membrane contained increasing amount of PS: 0 mol% (black squares), 5 mol% (red squares), 10 mol% (green squares), and 20 mol% (blue squares). The PIP2-donating membrane contained 5 mol% of PIP2 (solid squares), or both PIP2 and PI4P, 5 mol% each (empty squares). **(B)** Temporal evolution of the normalized cross-correlation amplitude upon Osh6 addition corresponding to the PIP2 biosensor release from the PIP2-donating LUVs. The PIP2-containing LUVs (5 mol%) were mixed with LUVs composed of the following: i) POPC: black squares, ii) POPC/PS (20 mol% of PS): red squares, and iii) POPC/PI4P (5 mol% of PI4P): blue squares. The presented data represent the trends that were observed in minimum three independent experiments.

The transport experiment consists of two steps: i) the extraction of the lipid from the donor membrane and ii) the insertion of the cargo lipid in the accepting membrane. These two processes cannot be fully separated when the label is only in the accepting membrane. However, this approach can be modified and the label can be also placed in the donor membrane in a separate experiment, and the extraction of PIP2 from the donor membrane can be monitored upon addition of the transporter (Figure 6B). The combination of the two outcomes allows us to distinguish whether PIP2 was transported to the acceptor membrane or whether it stayed bound to the transporting protein. If Osh6 is added solely to the PIP2-containing vesicles, the PIP2 level in the donor membranes significantly drops (Figure 6B, dotted curve). The drop approximately corresponds to the amount of added protein, that is, Osh6 binding capacity was around 80% of the available PIP2). When PC LUVs were added, all PIP2 was removed from the donor membrane (Figure 6B, black curve). However, there is only a little of PIP2 at the accepting membrane (Figure 6A). Altogether, our data show that PIP2 remains bound to Osh6. If PIP2 LUVs are combined with 20 mol% PS-containing LUVs, the extraction significantly slows down as PS competes with PIP2 for the lipid-binding pocket, and in the end, PS is replaced by PIP2 (red curve). Eventually, we combined PIP2-containing LUVs with PI4P-containing LUVs (blue curve); even though it does not correspond to the real biological situation, it helped us see that PIP2 extraction is blocked even more effectively with PI4P as it is a stronger Osh6 binder. PI4P however cannot be replaced by PIP2 and thus stays bound in the pocket.

These experiments revealed the relative tendency to bind the three ligands probed and the consequences for the Osh6-mediated lipid transport.

## Discussion

In our experiments, we have focused on understanding the biophysical determinants of the Osh6-mediated transport of PS. We reconstructed this process in the minimalistic system of artificial lipid bilayers. In our approach, we explored the process as it relates to the three individual participants of the transport: PS, PI4P, and PIP2. For detection of lipid movements, we have employed several lipid biosensors and characterized the range of their dynamic responses in each of the experimental conditions. This is an important consideration as it allows for reasonable planning and interpretation of the experiments.

We first focused on the relative binding affinities of the identified cargo molecules, that is, on the competition of these ligands for the Osh6 binding pocket. The strongest binder is PI4P, which can only be replaced by great excess of PS. Our experiments show that PI4P significantly inhibits the PS transport. This is a critical finding explaining how high PS levels in the PM can be maintained. Since PI4P competitively inserts into the Osh6 binding pocket, it prevents PS transport to be directed back to ER. Binding of PIP2 and PS seems to be of a similar scale in affinity but perhaps of a different mechanism. While PIP2 can affect the PS transport only slightly (Figure 1), PS excess is necessary for PIP2 replacement in the binding pocket of Osh6 (Figure 6A), that is, both the ligands compete for Osh6 with approximately equal chances; alternatively, PIP2 uses a different binding mode to the transporter. The PM contains both PIP2 and PI4P (van Meer et al., 2008); therefore, it is more likely that Osh6 will be occupied by PI4P, and thus PIP2 probably does not significantly contribute to the PS transport in the cell, or it modulates the transport by having impact on the lid opening for instance.

Second, we addressed the impact of the Sac1 phosphatase on Osh6 transport function. The presence of Sac1 on the surface of donor membranes restores the PS transport impeded by the presence of PI4P in the acceptor membrane. Apparently, PI4P must be removed at the PS-donating LUVs to continue the transport. We observed that i) PI4P reaches its target membrane prior to its hydrolysis (Figure 3) and ii) that PI4P hydrolysis by Sac1 was subjected to the presence of negatively charged lipids in the accepting membrane, specifically PS or PI (Figure 4). These findings together with the orientation of the Sac1 active site with respect to the negatively charged membrane (Figure 5) show that PI4P is transported and hydrolyzed in two independent steps, that is, it is not hydrolyzed while residing in the Osh6 binding pocket.

Third, we confirm that Sac1 requires PS or PI as an allosteric activator. This was observed previously, and our *in vitro* reconstitution system was able to recapitulate this observation (Zhong et al., 2012) and evaluate the PS or PI presence on the ER surface as a prerequisite of the PS transport process.

**FIGURE 7:**
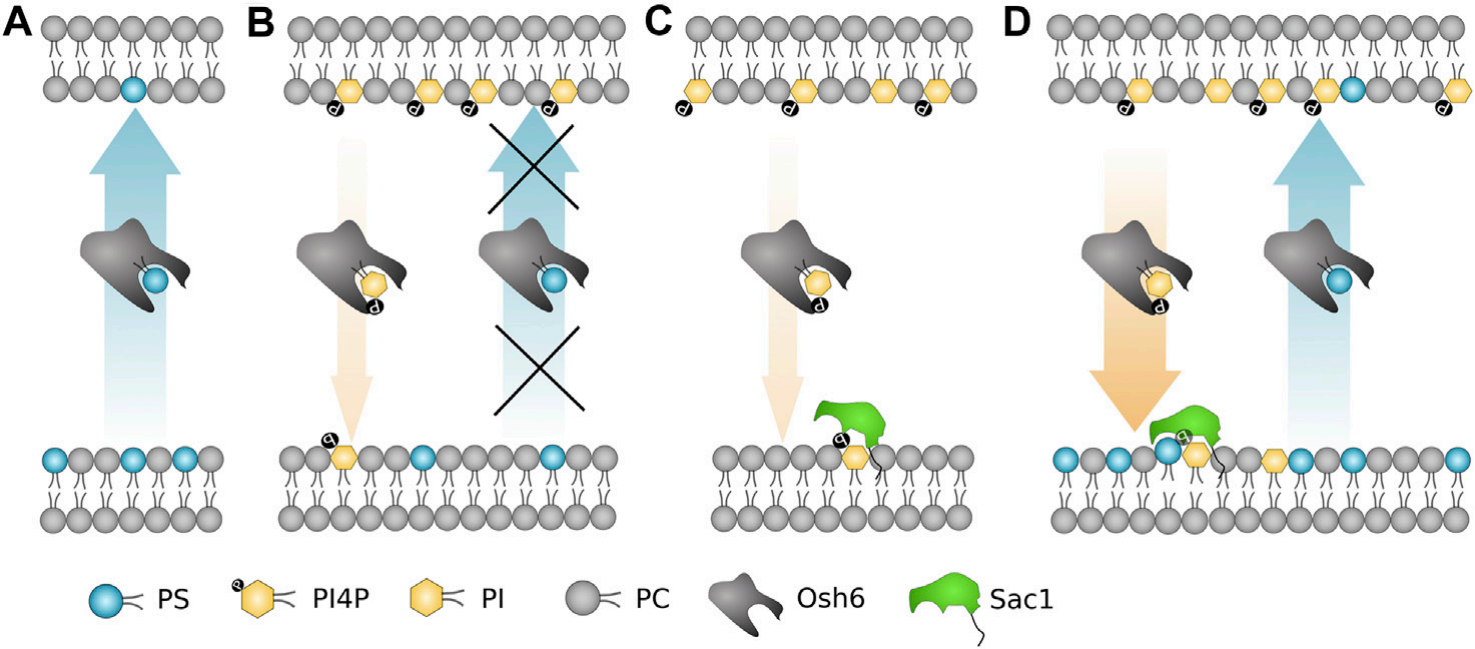
Schematic summary of the most important biophysical determinants for the Osh6 transport. **(A)** PS is transported along the gradient spontaneously. **(B)** If PI4P is present in the PS-accepting membrane, Osh6 binding pocket is occupied by PI4P and PS transport is inhibited. **(C)** Sac1 fails to dephosphorylate PI4P if no negatively charged lipid is present in the PI4P-acceptor membrane. **(D)** Both the PS and PI4P transports are occurring if the PI4P release to the acceptor membrane is synchronized with the Sac1 action, which is subjected to the PS (or PI, PG) presence in the acceptor membrane.

To sum up (Figure 7), the most important biophysical determinants of the transport revealed in our study are i) strong PI4P–Osh6 binding preference, which disables the PS transport along its concentration gradient; ii) Sac1 activity in the PS donor/PI4P acceptor membrane that removes transported PI4P; and iii) PS and PI activation of Sac1 in the donor membrane, that is, in the ER.

In the study of von Filseck et al. (2015a), the PS/PI4P exchange mechanism is proposed. Our study in agreement with that shows that all the investigated lipids are to a small extent transported spontaneously along the concentration gradient without a need for exchange. However, our experiments proved the strong tendency of Osh6 to transport PS along its concentration gradient with no need of exchange. In addition, we showed that PI4P instead of enhancing of the PS transport along its concentration gradient, it suppresses it. This suppression can be compensated by Sac1 action on the accepting membrane. Altogether, our data explain the directionality of the PS transport and provide rationale for the absence of the backward PS shuffling in the yeast membranes.

Recently, a study emphasizing a strong impact of the aliphatic chains on the transport scenario under non-exchange and exchange conditions (Ikhlef et al., 2021) appeared. The study shows that PS transport can be both accelerated and decelerated with PI4P depending on the saturation of the aliphatic chains and thus on the ligand-binding affinity. It is however worth noticing that the lipid translocation from the membrane environment to the Osh6 binding pocket is not only subjected to the lipid–protein interaction but also to the lipid–lipid interactions that occur in the membrane from which it is pulled out. Therefore, it is not surprising that our experiments done in significantly less fluid bilayer compared to the previous study (POPC versus DOPC) in some aspects differ as the composition of the lipid phase also has an impact on the lipid propensity for extraction by the transporter.

Our study addresses biophysical determinants of the transport of a single domain protein. Most proteins of the family, however, also have other domains that target the proteins in-between the two membranes, mostly to the membrane contact sites. Usually, the PH domain recognizes the PM, and the FFAT motif binds the VAP proteins in ER, or alternatively, ORP proteins have a transmembrane helix that anchors them directly to ER (Raychaudhuri and Prinz, 2010). *In vitro*, Osh6 transports the cargo molecules just as a result of their binding preferences and of a cooperation between the transporter and Sac1. *In vivo*, it requires Ist2, a membrane tether that localizes it, similarly as the other domains in more complex ORPs, to the membrane contact sites where it acts. Ist2 does not only localize the transporter but this interaction has also been shown to be critical for the PS transport as such and for the lipid metabolism (D'Ambrosio et al., 2020; Wong et al., 2021).

## Data Availability

The original contributions presented in the study are included in the article/Supplementary Material; further inquiries can be directed to the corresponding author.
