# Supplementary material for: Osh6 Revisited: Control of PS Transport by the Concerted Actions of PI4P and Sac1 Phosphatase
Source: Front Mol Biosci. 2021 Oct 12;8:747601. doi: 10.3389/fmolb.2021.747601 (PMC8546167; doi:10.3389/fmolb.2021.747601)
Supplement: Supplementary file 1 [file datasheet1.pdf]

**Supporting information**

Calibration of the biosensor responses

The dynamic range of the response of individual biosensors used in this study were monitored in separate experiments. The calibration was done under the same conditions we used for the transport experiments.

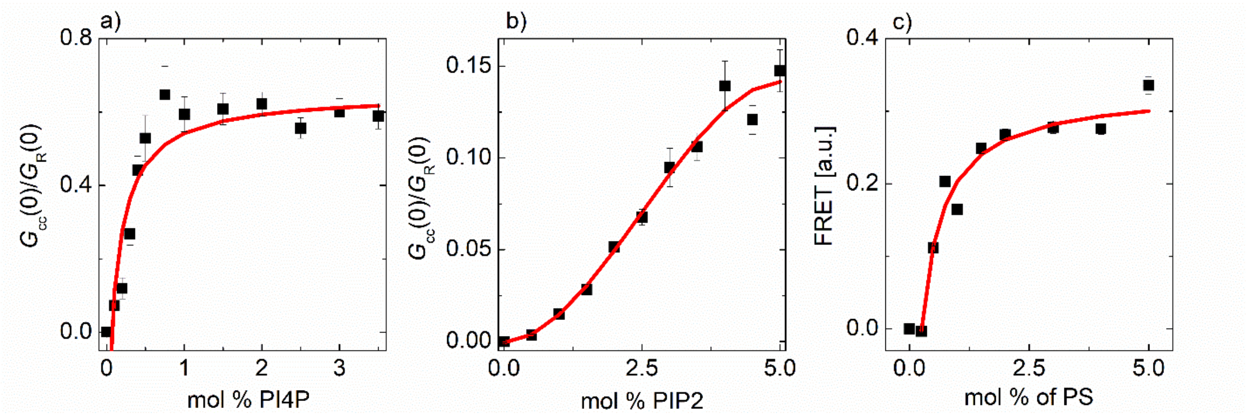


Figure S1. Response of the used techniques to the amount of the lipid of interest in the membrane of LUVs sensed by its biosensor. The calibration was acquired under the same conditions we used for the transport experements. a) FCCS read-out $G_{cc}/G_R$ as a function of the molar fraction of PI4P in the membrane, b) FCCS read-out $G_{cc}/G_R$ as a function of the molar fraction of PIP2 in the membrane, a) FRET read-out as a function of the molar fraction of PS in the membrane.

The following table shows apparent dissociation constants for the biosensors:

| Biosensor/lipid | $K_D$/μM |
|---|---|
| SidC/PI4P | 0.014 +/- 0.005 |
| $C2_{granuphilin}$/PIP2 | >>1 |
| $C2_{Lact}$/PS | 0.62 +/- 0.13 |

Table S1. Apparent dissociation constants for individual biosensors, the error stands for 95 % confidence interval.

Evaluation and use of the FCCS data

Fluorescence crosscorrelation spectroscopy (FCCS) is a microscopy technique that suits for monitoring interactions of fluorescently labeled species. It uses the confocal microscope to focus excitation light into a diffraction limited spot. Motion of fluorescently labeled particles through the focus translates into fluctuations in the detected signal. These fluctuations refer on the number of molecules in the focus and the speed of their diffusion through. If additionally, two differently labeled species are excited by two different overlapping laser beams and the resulting signal is spectrally split and detected on two detectors,

the relatedness of the two fluctuation traces refers on the mutual interaction. The relatedness is quantified by so called cross-correlation function $G_{cc}(t)$:

$$G_{\mathrm{cc}}(\tau) = \frac{\langle \delta I_{\mathrm{G}}(t) \delta I_{\mathrm{R}}(t+\tau) \rangle}{\langle I_G \rangle \langle I_R \rangle}, \quad (1)$$

where $\delta I$ is a fluctuation of the green and the red signal, the brackets stand for temporal averaging. The correlation decays with time $\tau$, which is the time lag between the green and the red signal. The interacting species are monitored at $\tau = 0$, as at zero time lag, diffusion does not cause any decay of the correlation and its amplitude refers on the concentration of the interacting participants.

In the case of the experiments done here, the red labeled species are the LUVs containing the red fluorescent marker DiD. The green species are the CFP labeled biosensors for various lipids (PI4P, PIP2). During the transportation the lipid of interest is moved from the unlabeled LUVs to the red labeled LUVs and the biosensor goes along. For the crosscorrelation amplitude it holds:

$$G_{\mathrm{cc}}(0) = \frac{c_{\mathrm{R}} \sum_{n=0}^{N} f_{\mathrm{G}_n\mathrm{R}} \cdot n}{V_{\mathrm{eff\,GR}} c_{\mathrm{R}} \left( c_{\mathrm{R}} \sum_{n=0}^{N} f_{\mathrm{G}_n\mathrm{R}} \cdot n + c_{\mathrm{G}} \sum_{m=0}^{M} f_{\mathrm{G}_m} \cdot m \right)}, \quad (2)$$

where $c_R$ and $c_G$ is concentration of the red labeled LUVs and the LUVs, from which the biosensor is moved by following the lipid transport, respectively. $f_{GnR}$ is the fraction of red LUVs bearing $n$ molecules of the CFP-biosensor. $f_{Gm}$ is the fraction of the unlabeled LUVs with $m$ molecules of the CFP-biosensor. $V_{eff\,GR}$ is the effective volume of the two overlapping foci.

In order to keep the transport monitoring parameter independent of the red labeling (the concertation of the red dye can vary due to pipetting inaccuracies, for example), we always follow the ratio between the crosscorrelation and the red autocorrelation amplitude $G_R(0)$ ($G_{\mathrm{R}}(0) = 1/(V_{\mathrm{eff\,R}} C_{\mathrm{R}})$). Eventually, the FCCS transport referring read-out is:

$$\frac{G_{\mathrm{cc}}(0)}{G_{\mathrm{R}}(0)} = \frac{V_{\mathrm{eff\,R}}}{V_{\mathrm{eff\,GR}}} \cdot \frac{c_{\mathrm{R}} \sum_{n=0}^{N} f_{\mathrm{G}_n\mathrm{R}} \cdot n}{\left( c_{\mathrm{R}} \sum_{n=0}^{N} f_{\mathrm{G}_n\mathrm{R}} \cdot n + c_{\mathrm{G}} \sum_{m=0}^{M} f_{\mathrm{G}_m} \cdot m \right)}. \quad (3)$$

It consists of the volume ratio of the red focus and the overlap of the two foci (geometry of the beams) and from the contribution of biosensor distribution between the labeled and unlabeled LUVs.

The distribution of the biosensor molecules among the given type of LUVs is assumed to be Poissonian. Fig. S2a illustrates how the biosensor molecules are distributed between the two different LUV types at 5, 25 and 60 % of the biosensor translocation. Independent of the ratio of the two types of LUVs and the total amount of the biosensor, the amplitude ratio $G_{cc}/G_R$ is directly proportional to the fraction of the translocated biosensor (Fig. S2b – red line).

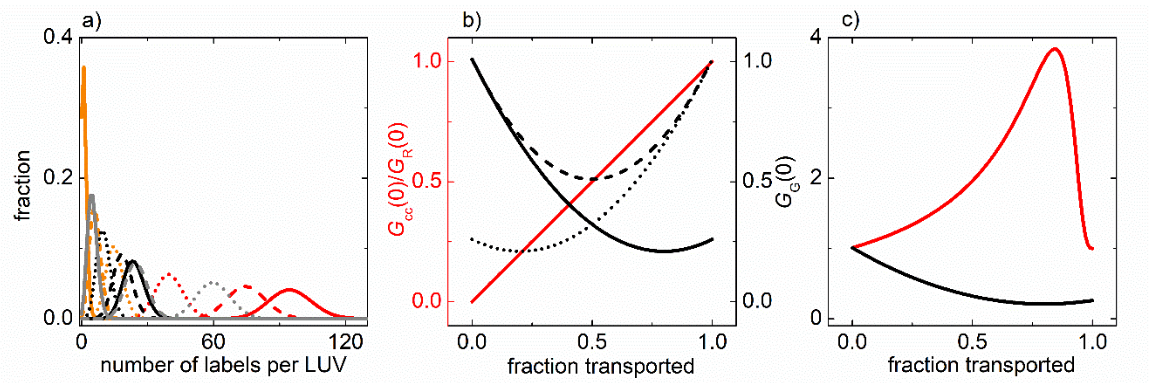


Figure S2. Illustrative explanation of use and meaning of amplitudes of auto- and crosscorrelation functions. a) Poisson distributions of the biosensor on donating and accepting vesicles at two different molar ratios of LUVs: donating/accepting (1/4) – red/orange curves; donating/accepting (4/1) – black/grey curves. The lines show different fractions of biosensor that has moved from the donating to the accepting LUVs: 5 mol % – solid lines, 25 mol % – dashed lines, 60 mol % – dotted lines. b) Red: direct proportionality between the lipid transport read-out $G_{cc}/G_R$ and the fraction of the translocated transporter (independent on the LUV ratio). Black: Dependence of the autocorrelation function amplitude $G_G$ of the fluorescetly labelled biosensor on the fraction of the biosensor transpoted from the donating to the accepting LUVs. The LUVs were in various ratios: donating/accepting (1/4) – solid line, donating/accepting (1/1) – dashed line, donating/accepting (4/1) – dotted line. c) Black line corresponds to the black solid line from S1b. The red line shows the behavior of the $G_G$ amplitude in the same situation if additionally PI4P is dephosphorylized by Sac1 and the biosensor is released to the solution.

Additional FCCS parameter that refers on the transport is the amplitude of the green autocorrelation function. It follows the formula:

$$G_{\mathrm{G}}(0) = \frac{c_{\mathrm{R}} \sum_{n=0}^{N} f_{\mathrm{G}n\mathrm{R}} \cdot n^2 + c_{\mathrm{G}} \sum_{m=0}^{N} f_{\mathrm{G}m} \cdot m^2}{V_{\mathrm{eff\,G}} \left( c_{\mathrm{R}} \sum_{n=0}^{N} f_{\mathrm{G}n\mathrm{R}} \cdot n + c_{\mathrm{G}} \sum_{m=0}^{M} f_{\mathrm{G}m} \cdot m \right)}, \tag{4}$$

$V_{\mathrm{eff\,G}}$ is the volume of the green focal region. Fig. S2b (black curves) depicts three curves for different ratios of red and unlabelled LUVs. All three are parabolic showing that first, a decay is observed when the biosensor molecules start occupy more LUVs, and second, they start prevailing at the target LUVs, i.e. the amount of labelled particles drops which is accompanied by the increase in $G_G$ amplitude.

We have also used $G_G$ to visualize the transport of PI4P followed by its dephosphorylation in the target LUVs. If PI4P is only transported and the dephosphorylation does not occur, decay in $G_G$ can be observed (black solid curve in Fig. S2b and S2c). However, if the dephosphorylation of Sac1 occurs upon the transport, the biosensor is not transferred to LUVs but instead is released to the solution, this is manifested by the increase in the $G_G$ amplitude (red curve in Fig. S2c).
